# Dividend policy issues in the European pharmaceutical industry: new empirical evidence

**DOI:** 10.1007/s10198-022-01510-5

**Published:** 2022-08-26

**Authors:** Tobias Basse, Christoph Schwarzbach, J.-Matthias Graf von der Schulenburg

**Affiliations:** 1 Norddeutsche Landesbank (NORD/LB), Friedrichswall 10, 30159 Hannover, Germany; 2 Touro College Berlin, Am Rupenhorn 5, 14055 Berlin, Germany; 3 Institute of Information Systems Research, Gottfried Wilhelm Leibniz University Hannover, Koenigsworther Platz 1, 30167 Hannover, Germany; 4 Institute for Risk and Insurance, Gottfried Wilhelm Leibniz University Hannover, Otto-Brenner-Straße 7, 30159 Hannover, Germany

**Keywords:** Dividend policy, Granger causality, Pharmaceutical industry, Health care firms, Research and development expenditures, Litigation risk, G35, L65, K41, O32, O34

## Abstract

This paper examines dividend policy issues in the European pharmaceutical industry. This sector is of particular interest because of the high research and development expenditures and the associated risks characterizing the business models of many firms in this industry. In fact, from the perspective of corporate finance theory, this is a particular challenge for the managers of these corporations that may also have implications for the dividend policy implemented by the firms forming this sector. Moreover, the level of internal financing and litigation risks also seem to be high in the pharmaceutical industry. These facts could also affect the payout policy of the firms. Employing techniques of time series analysis, there is no evidence for dividend signaling and clear evidence for dividend smoothing in the European pharmaceutical industry. Given that dividend increases under certain assumptions can negatively affect the firms' ability to finance new investments in general and research and development projects in particular, these results of our empirical investigations could be described as highly plausible.

## Introduction

Trying to explain why firms decide to pay dividends has created significant problems for financial economists. In fact, the existence of dividend payouts has been called puzzling, and empirical researchers in the field of corporate finance have stressed that there is no consensus to answer the question of why firms pay dividends (see, most importantly, [1, 2]). In spite of numerous research efforts, there still seems to be no clear picture (see, amongst others, [3, 4]). Baker et al. [5] have stressed that the dividend policy preferred by the managers of a corporation can differ substantially from one firm to another. Consequently, it can be argued that dividend policy issues should be analyzed focusing on firm-specific factors. However, Van Caneghem and Aerts [6] have noted that firms belonging to one industry tend to imitate the dividend policy of their peers. Thus, examining dividend policy issues following a sectoral approach also should be seen as a useful empirical research strategy. In fact, this approach has become quite popular recently (see, for example, [7, 8]).

We try to add to this literature by examining dividend policy issues in the European pharmaceutical industry. This sector is of particular interest because of the high research and development (R&D) spending that characterizes the business models of many firms in this industry (see, for example, [9]). From the perspective of corporate finance theory, this is a particular challenge for the managers in these corporations that may also have implications for the dividend policy followed by the firms comprising this sector. More specifically, Tirelli and Spinesi [10] have argued convincingly that R&D investment is more difficult to finance due to information asymmetries between insiders (which means managers) and outsiders (especially bond investors). They have noted that R&D-intensive firms, therefore, might want to rely more strongly on internal funding sources and equity than on debt financing. Accepting this point of view, there are clear implications for the dividend policy of these firms, because compared to other business enterprises, it could be very costly for R&D-intensive firms to pay out funds to their investors as dividends. Moreover, as will be discussed in more detail later on, litigation risk seems to be comparably high in the pharmaceutical industry. This might also have consequences for the payout policy of firms. In fact, there is the idea that litigation risk might affect the payout policy of firms (see, most importantly, [11, 12]). Thus, there are at least two very good reasons why the dividend policy of firms belonging to this economic sector could be of particular interest. Moreover, these two reasons are also closely linked to each other. As will be discussed later on in more detail, litigation risk and R&D expenditures may, for instance, be highly related due to patent disputes. Furthermore, the issue of product liability might also be of some importance in this context. We use techniques of time series analysis [13, 14] to investigate this issue. To our knowledge, this has not been done before. More specifically, our empirical research strategy is based on the approach of Goddard et al. [14] and uses the concept of Granger causality (see [15, 16]). To be more precise, we analyze dividend payouts and corporate earnings data from the European pharmaceutical industry and employ the approaches suggested by Johansen and Toda and Yamamoto [17, 18] to test for Granger causality (respectively, Granger non-causality).

The present paper is structured as follows. Section “Dividend policy: relevant or irrelevant?” briefly reviews the literature on dividend policy issues in general. Section “Research and development expenditures and pricing in the pharmaceutical industry” then highlights the importance of R&D expenditures for the pharmaceutical industry and tries to explain possible consequences from the perspective of corporate finance theory. Based on these thoughts, we discuss the relevance of litigation risk for the payout policy of firms in section “Dividend policy and litigation risk”. Following this vital part, some methodological issues are explained in section “Data and some methodological issues”. The data examined is also introduced here. Section “Empirical analysis” then reports the results obtained from our empirical investigations. Section “Conclusion” finally summarizes and provides suggestions for future research .

## Dividend policy: relevant or irrelevant?

Miller and Modigliani [19] have argued that under certain conditions, a firm's dividend policy is irrelevant for the stock price and, therefore, for the wealth of the owners of this corporation. They assume perfect capital markets with rational investors, a given investment policy of the firm under investigation and the absence of taxes. In this simplified model world, higher dividend payments would simply result in lower capital gains for equity investors. Consequently, the dividend policy followed by the firm would not be relevant from an economic point of view, assuming that investors do not prefer dividends to capital gains or vice versa. This is the so-called dividend irrelevance theorem which is the theoretical basis for the already discussed proposition that there is a dividend puzzle (see, for example, [1, 20]). In this context, Baker and Weigand [21] have, in fact, stressed the high importance of Miller and Modigliani [19]. Given that the managers of firms use resources to formulate a dividend policy that is presumed to be beneficial in some way for shareholders, this puzzle should be solved, because accepting that dividend payouts really are irrelevant would otherwise imply that these corporations do waste time and money thinking about their dividend policy. Taxes may be of relevance at this point. In this regard, it must be noted that, at least in some countries, dividends are taxed more heavily than capital gains (see, for example, [1, 21]). In spite of this tax disadvantage, many firms still decide to pay dividends. Thus, it could be argued that there are circumstances where the presence of taxes makes the existence of dividend payouts even more puzzling. While Bernheim [22] has developed a theoretical model to explain this phenomenon, many open questions remain.

It has to be noted that there has also been criticism of the assumption that dividend payments are of no economic relevance. Most importantly, Baker et al. [5] have stressed that the presumed irrelevance of the dividend policy followed by a firm becomes more debatable once researchers leave the idealized world of economic theory. In any case, the real world is more complex, and now there seems to be some kind of consensus in the field of corporate finance that agency theory ought to be of some importance to answer the question of why firms decide to pay dividends (see, for instance, [5, 23]). In fact, distributing funds to the investors can be an effective way to overcome agency problems between corporate insiders—in other words, a firm's management—and the shareholders who are outsiders. Aivazian et al. [24], for example, have argued convincingly that dividend payments can force a firm to interact with current and potential new investors more frequently. Simply put, paying dividends can force a firm's management to obtain capital from external sources more often to finance new investment projects. In order to raise additional funds, a corporation is required to give more information to market participants. Consequently, the process of raising new capital can help to reduce agency costs. However, in this context, it must be taken into account that obtaining capital from external sources always also generates transaction costs. Thus, not all payout policy measures that reduce agency costs necessarily are beneficial for the shareholders.

It has also been argued that the managers of a firm as insiders could change the volume of the dividends paid by a corporation to provide private information to current and prospective investors (see, for example, [25, 26]). This interesting concept is called the dividend signaling hypothesis. Accepting this idea, the dividend policy followed by a firm might be helpful to mitigate information asymmetries between the management of a firm and other relevant economic agents. However, there is the fear that financial markets could interpret dividend cuts or omissions as very negative signals and a sign that managers (as insiders) expect major future problems. This would be a particular problem when there is the danger of an overreaction of the stock price to the new information (see, amongst others, [27, 28]). In this context, Lintner [29] has argued that the managers of a firm could have an incentive to try to prevent the need for erratic changes to dividend payouts. Therefore, firms might want to avoid the possible troubles associated with the announcement of dividend reductions by only gradually increasing their dividend payments. More specifically, given that dividends are paid from corporate earnings, the volume of dividend payouts should only be increased when there is a high likelihood that the stream of expected future earnings will at least in normal times be sufficiently strong to shoulder the increased financial burden from higher dividend payments. Following this strategy would ensure that dividend reductions remain an exception. This is the dividend smoothing hypothesis (see, for example, [14, 20]). Goddard et al. [14] have argued convincingly that both the dividend signaling hypothesis and the dividend smoothing hypothesis assume the existence of a close relationship between earnings and dividends but have also noted that timing issues have to be kept in focus. In fact, according to the dividend signaling hypothesis, dividend payouts should lead corporate earnings, and according to the dividend smoothing hypothesis, corporate earnings ought to lead dividends.

As already noted, it is very common in the theoretical literature to assume that the investment policy of a firm is given. However, this could be a problem in some cases. In fact, DeFusco et al. [30] have argued convincingly that under certain conditions—for example, when capital markets are constrained or incomplete—increases to dividend payouts could limit the ability of a firm to finance new investment projects. They have argued that those firms should consider alternative ways to signal information about future earnings to their investors and other interested parties. In general, managers ought to carefully evaluate the impact of dividend increases on their investment policy and the future earnings of their business enterprises. This could, for example, have significant implications for the pharmaceutical industry. In fact, Audretsch and Weigand [31] have argued convincingly that knowledge-based economic activities tend to be subject to higher degrees of uncertainty and asymmetric information. Therefore, access to external sources of finance for these activities might be more difficult for firms.

Bhagwat and DeBruine [32] have argued that advertising is of particular importance in the pharmaceutical industry. This might also have effects on financing decisions. In fact, Klein and Leffler [33] have shown that investments in the reputation of business enterprises can have an effect on their future behavior. Thus, expenditures for more advertisement might not only help to increase the future business activity of pharmaceutical firms but could also help to signal information to financial market participants. Therefore, providing more resources to the advertising budget might be seen as a more efficient way of signaling for these business enterprises compared to dividend increases.

Patents may also matter. Hottenrott et al. [34], for example, have suggested using patents as signals to investors to cope with the information asymmetries between the management of a firm and potential lenders and investors. At this point, however, a sort of vicious circle could become apparent because Schroth and Szalay [35] have shown that the availability of financial resources clearly helps firms to win patent races. They have presented empirical evidence from the US pharmaceutical industry that points into this direction. Both the own cash reserves and the cash reserves of the rivals appear to play a role here. This empirical finding clearly has some implications for the payout policy of the firms under investigation in this empirical study and also supports the idea that the sectoral approach taken here with a focus on the pharmaceutical industry is likely to be particularly worthwhile.

In any case, it is clear that there are some hypotheses discussed in the literature with respect to the financing decisions (and as a result to the payout policy) of firms in general that could be of relevance at this point (see, for example, [36, 37]). Moreover, additional considerations could be valid for the pharmaceutical industry in particular, because this economic sector might be quite special with regard to its financing decisions (see, for instance, [38, 39]).

Sasidharan et al. [40] have presented data from India—where the pharmaceutical industry plays a special role—that clearly show a relationship between internal cash flows and R&D activity and a great reluctance of firms to finance R&D investments using equity issuance. Of course, this would violate one central assumption of the hypothesis that dividends are irrelevant (see, most importantly, [19]). At this point, it is certainly of relevance to note that Ghosh [41] has reported that the liberalization of the financial markets in India seems to have reduced the financing constraints in this country—and that the size of the firms also plays a role with regard to this issue. In this context, it is of particular importance that the empirical findings reported by Nylund et al. [37], who have examined data from 146 large European firms over 10 years, seem to indicate very clearly that external financing in the form of debt reduces the focus on innovation in profitable firms. Moreover, Czarnitzki et al. [36] have noted that it is evident that there are financing constraints for investments in R&D activities due to capital market imperfections and the unique features of these investments. They have argued that it might be necessary to examine research and development separately. As a matter of fact, they have shown that research investment is even more sensitive to the liquidity situation of a firm than the development activities of business enterprises. This finding implies that firms have to rely even more strongly on internal funds to finance research investments compared to their development activities. Consequently, the role of the R&D expenditures in the pharmaceutical industry will now be examined in some detail.

## Research and development expenditures and pricing in the pharmaceutical industry

Since pharmaceutical markets are rarely subject to free pricing due to a variety of economic characteristics (e.g. arising from information asymmetries and external effects), the magnitude of R&D costs and, in some cases, other cost categories of the pharmaceutical industry (e.g. production, marketing, and distribution costs) is a much-discussed factor, especially with regard to the perceived appropriateness of pharmaceutical prices and the resulting expenditures for the health care system.

**Fig. 1:**
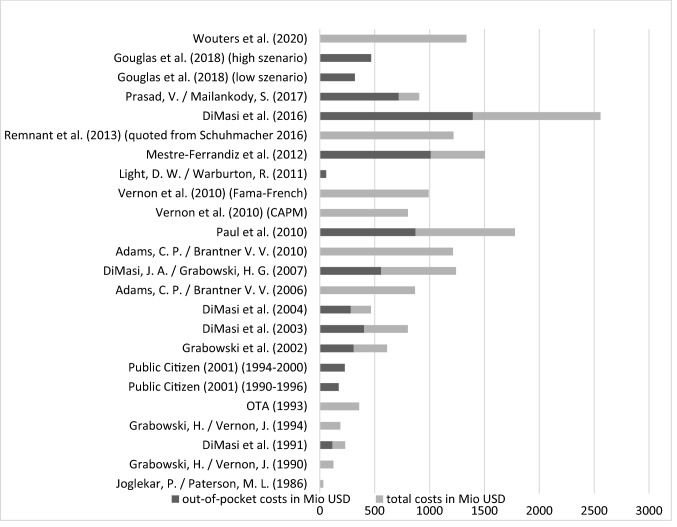
Different published estimates for the average costs of pharmaceutical R&D. *Source*: [42, 43, 45, 46, 53, 56, 59, 61, 99–110]

A widely cited study by DiMasi et al. [42] calculates the R&D costs for a drug up to approval to be over 800 million USD. Paul et al. [43] even estimate the costs at around 1.8 billion USD. Morgan et al. and Schuhmacher et al. [44, 45] provide surveys on this issue. A representation of the most important study results on R&D costs can be found in Fig. 1, although it has to be mentioned that their comparability with one another is limited [46].

The main cost drivers in pharmaceutical R&D are the prolonged duration of the process [47], the probability of market approval which is closely related to the high uncertainty of the R&D projects [42, 43, 48–50], the capital, respectively, opportunity costs of the relatively long-term investments, and, of course, the costs of conducting the research and the necessary studies. Especially over time, changes in these factors can be observed, for example, due to medical inflation or higher regulatory requirements [45, 50].

The values mentioned above, and in particular, the influential study by DiMasi et al. [42], are often criticized as being too high (see, for example, [51]). The arguments against these studies focus, among other things, on the financial support of the conducting research institute by the pharmaceutical industry [52, 53], the possibly overstated development times [54], the lack of transparency of the underlying data [55], the calculation on a pre-tax basis [42, 52, 53, 56, 57], the use of government-funded basic research by the pharmaceutical industry [58], the unclear separation between the costs of R&D and other expenses [52], and the significant influence of the interest rate on the results [59–61].

Grabowski and Vernon [62] identify two key determinants for the investment in R&D projects—the expected return on R&D and the availability of Cash Flows. In contrast, Bhagat and Welch [63] find no relation between R&D expenditures and operating cash flows but a positive relationship between R&D spending and 2-year lagged stock returns for European companies. The returns on marketed pharmaceutical products can be highly skewed as very few drugs report returns that exceed the calculated R&D costs (see, [62]). In the long term and on average, the prices must, among other things, compensate for R&D expenditures over the course of their product life cycle. This makes drug pricing vital to the business model of the pharmaceutical industry. Abbott and Vernon [38] show for the US by means of a Net Present Value-based prospective micro-simulation that price regulations and corresponding price cuts will in turn reduce the incentives for early-stage R&D investments. Additionally, Giaccotto et al. [64] find a positive relationship between real drug prices and R&D spending.

**Fig. 2:**
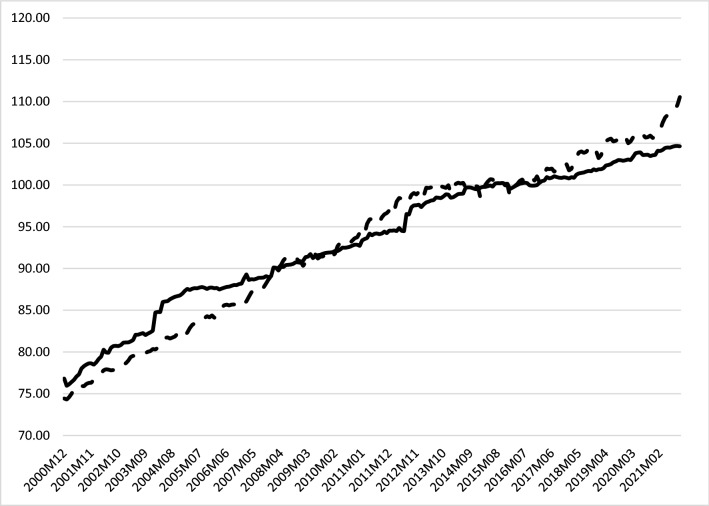
Harmonized Index of Consumer Prices (HICP): All Items (dotted line) and Pharmaceutical Products (continuous line) for the European Union (27 from 2020) (2015 = 100). *Source*: Data retrieved from Eurostat [111]

Within the health care systems, the proportion of pharmaceutical spending and drug prices increase over time (see, e.g., [9]). The prices are mainly regulated—be it directly or indirectly—by the respective governments, counteracting, among other things, the monopolies established by patent protection for the drugs and a demand-side characterized by inelasticity and moral hazard. Drug expenditure in proportion to total health expenditures as well as drug expenditure per capita have grown in nearly all key EU countries in the last 2 decades [65, 66]. At this point, the macroeconomic phenomenon of inflation also has to be taken into account. In fact, as represented in Fig. 2, consumer prices have risen in Europe over the previous years.

Higher drug prices obviously do affect health care spending and, therefore, could become a problem for public policymakers. This might have major implications for the business models and activities of the firms that belong to the pharmaceutical industry. More specifically, higher drug prices could also result in more regulation and government control of the business activities of the firms in this sector of the economy (see, most importantly, [9]). In fact, policymakers might even want to limit the ability of the pharmaceutical industry to increase drug prices. This is a very special type of risk from the perspective of the theory of corporate finance that could also have implications for the dividend policy of the firms that belong to this sector of the economy. This potential scenario might be an argument for greater caution by corporations when it comes to dividend increases. In this context, it is also important to keep in mind that there is empirical evidence that inflation appears to generally increase dividend payouts of firms over time (see, most importantly, [13]).

A potentially vital additional side aspect (namely patent litigation) of the R&D efforts in the global pharmaceutical industry that also can affect the dividend policy of firms will be discussed in some detail in the next section.

## Dividend policy and litigation risk

Litigation risk might also matter for the payout policy of firms. There seems to be no doubt that litigation risk can affect several corporate policies (see, for example, [67, 68]). Thus, it should come as no surprise that the existence of major legal risks could also affect the dividend policy that a firm decides to follow. As a matter of fact, Malm and Kanuri [12] have argued convincingly that the potential financial damage from litigation may complicate the ability of a company to raise external capital and that, as a consequence, sued firms might prefer a more conservative dividend payout policy to distribute fewer funds to shareholders. Given that Yuan and Zhang [69] as well as Arena [11] have reported some empirical evidence indicating that companies that face a higher risk of litigation sustain higher debt costs, this behavior could indeed make sense. In this context, Arena [11] has stressed that litigation risk has a negative effect on credit ratings. In any case, given that banks seem to demand higher interest rates for loans made to sued firms to compensate the lender for the legal risks that might hurt the borrower's future earnings, it could be a good strategy for these corporations to pay less dividends. There are several ways in which litigation risk can have negative effects on corporate earnings. First, of course, the legal fees should be mentioned in this context (see, e.g., [70, 71]). But there obviously are also other relevant costs. The potential reputational loss from litigation, for example, can hurt profits (see, for instance, [33, 72]). Arena and Ferris [73] have discussed the determinants of litigation risk in some detail from the perspective of corporate finance theory. It has already been noted that dividends are paid from corporate earnings and that—because litigation risk clearly can affect the profitability of a firm—companies that face major lawsuits might therefore be less willing to pay dividends. Even the threat of being forced to go to court may affect a firm's payout policy. Therefore, it is probably no major surprise that the empirical findings of Malm and Kanuri [12], who have examined data from the United States, seem to indicate that pending lawsuits reduce the willingness of firms to pay dividends. At this point, it should be noted that Reddemann et al. [74] have argued that dividend smoothing is dividend signaling with precaution. Consequently, it might be assumed that firms facing litigation risk could prefer a payout policy guided by the principle of dividend smoothing to a policy that follows the concept of dividend signaling.

Examining data from the United States, Kim and Skinner [75] have shown that lawsuits cluster by sector and that healthcare firms are one of the industry groups that face the most lawsuits. Given that just a higher risk of being sued might already affect the payout policy of firms, this observation is important. Furthermore, it is also of relevance in this context that, as already noted, Van Caneghem and Aerts [6] have argued convincingly that firms belonging to the same sector of the economy tend to imitate the dividend policy of their competitors. Therefore, a sectoral approach to the empirical analysis of dividend policy issues clearly makes sense.

Regarding the pharmaceutical industry, the patent premium for very few compounds is very high, raising the average as a whole compared to other sectors [76]. From a regulatory perspective, the guaranty of patent protection is intended to achieve mainly two goals. On one hand, it ensures that society benefits from the invention, and, on the other hand, the incentives aim to promote further innovations [77]. Patents additionally reduce transaction costs [78]. Patent litigation also seems to play a significant role (see, for example, [79–81]), and the litigation numbers seem to increase over time [82]. Legal disputes often result in settlements of the parties and not necessarily in court decisions [82]. The legal challenges and the associated costs reduce the incentives for R&D expenditures for the pharmaceutical industry (see, e.g., [83, 84]).

Two other potential sources of additional expenses are, on the one hand, the possibility of plaintiffs to initiate preliminary injunction hearings [85] and, on the other hand, possible costs associated with product liability. For the latter, the number of cases and large payments has increased over time, and the reasons for litigations have broadened, at least in the US (see, for example, [59]). These increases are also reflected in, e.g. higher premiums and deductibles on the market for product liability insurance leading to more self-insurance by the pharmaceutical companies (e.g. increasing reserves or founding own insurers) [59]. The announcement of potential product liability issues or the actual filing of a lawsuit lead to significant losses of the firm's value, possibly including reputational effects, while competitors' firm values increase [86]. To safeguard against possible liability costs, companies could feel the pressure to increase costs and time for R&D further or even to avoid particularly high-risk research areas altogether [59]. Such a behavior of the firms could even have an influence on the medical and technical progress of the society as a whole.

## Data and some methodological issues

As already noted, the present paper examines data from the European pharmaceutical industry. More specifically, we analyze the dividend per index share and earnings per index share (EPS before extraordinary items) of the Bloomberg Europe 500 Pharmaceuticals Index. All companies that are a member of the broad Bloomberg Europe 500 Index and involved in the pharmaceutical sector are included in this equity index. We examine quarterly data. All time series under investigation are obtained from Bloomberg. The sample is Q1 2002 to Q4 2020.

Our empirical analysis is based on the concept of Granger causality (see [15, 16]). A time series *Y* is said to Granger cause the variable *X* if it contains information that helps to improve the forecastability of *X*. More specifically, the time series *X*_*t*_ is not Granger causing the time series *Y*_*t*_ if for all *n* > 0:

1 $$F(Y_{(t + n)} |\Omega_{t} ) \, = \, F(Y_{(t + n)} |\Omega_{t} - \, X_{t} ),$$

where *F* denotes the conditional distribution and the expression *Ω*_*t*_ – *X*_*t*_ describes all information that might be of relevance except for *X*_*t*_.

Given that we observe two possibly non-stationary variables that may be related to each other in a dynamic way—the existence of feedback effects, for example, cannot be ruled out—we employ the procedures suggested by Johansen and Toda and Yamamoto [17, 18] to test for Granger causality. Both techniques are based on the concept of vector autoregressions which has been pioneered by Sims [87]. This approach can adequately model the complex dynamic interaction among the time series under investigation here. More specifically, in Eq. (2), *Y*_*t*_ is a vector of (*n* × 1) endogenous variables, *A*_*i*_ are (*n* × *n*) coefficient matrices, and *ε*_*t*_ is a disturbance term (and also an (*n* × 1) vector):

2 $$Y_{t} = \, A_{1} Y_{t - 1} + \, A_{2} Y_{t - 2} + \cdots + \, A_{n} Y_{t - n} + \, \varepsilon_{t} .$$

If necessary, an (*n* × 1) vector of constants or seasonal dummy variables can be added to this model. Using the approach suggested by Toda and Yamamoto [18], a vector autoregression in levels is estimated even for non-stationary variables. This model includes p time lags and is extended by *q* additional surplus time lags to perform modified Wald tests to search for Granger causality where *q* is the highest order of integration of any variable that is considered in the model and p is the optimal number of time lags for the vector autoregression. The surplus lags ensure that the test statistic is asymptotically chi-square distributed. Thus, the model that is specified in Eq. (3) is estimated to test for Granger causality:

3 $$Y_{t} = \, A_{1} Y_{t - 1} + \, A_{2} Y_{t - 2} + \cdots + \, A_{p} Y_{t - p} + \, A_{p + q} Y_{t - (p + q)} + \varepsilon_{t} .$$

Again, constant or seasonal dummy variables might be added to this model. Moreover, assuming that the variables examined are non-stationary cointegration could be a phenomenon of economic relevance. Cointegration means that there is a linear combination of two non-stationary variables that is stationary (see, for example, [88, 89]). Finding cointegration among time series implies the existence of long-run equilibrium relationships among the variables examined. In this case, it is possible to employ an approach to analyze cointegrated systems that has been developed by Johansen (see, most importantly, [17, 90]). In fact, a vector error correction model (VECM) can be estimated to test for Granger causality. Rewriting Eq. (2) leads to:

4 $$\Delta Y_{t} = \, \left( {A_{1} {-} \, I} \right) \, y_{t - 1} + \, A_{2} Y_{t - 2} + \cdots + \, A_{n} Y_{t - n} + \, \varepsilon_{t} ,$$

5 $$\Delta Y_{t} = \, \left( {A_{1} {-} \, I} \right)\Delta y_{t - 1} \, + \, \left( {A_{1} + \, A_{2} {-} \, I} \right) \, Y_{t - 2} + \cdots + \, A_{n} Y_{t - n} + \, \varepsilon_{t}$$

and

6 $$\Delta Y_{t} = \Pi_{1} \times \Delta Y_{t - 1} + \Pi_{2} \Delta Y_{t - 2} + \cdots + \Pi Y_{t - n} + \varepsilon_{t} = \Pi_{i} Dy_{t - i} + \Pi y_{t - n} + \varepsilon_{t} ,$$

where

$$\Pi_{i} = {-}\left( {I{-} \, \sum\limits_{h\, = \,1}^{i} {A_{h} } } \right) \, ,$$

$$\Pi = {-} \, \left( {I{-}\sum\limits_{i\, = \,1}^{n} {A_{i} } } \right).$$

In all cases, Δ means first difference. It is also possible to include seasonal dummies, intercepts and deterministic trends in models of this type if necessary. Using this approach, *k* cointegration relationships among the variables examined are said to exist when the rank of the matrix Π is *k* < *m*. Two different likelihood ratio tests for the reduced rank of Π have been suggested by Johansen [17]. Here, the so-called trace test is employed:

7 $${\text{Trace}}\,{\text{Stat}} = {-}T\sum\limits_{i\, = \,k + 1}^{m} {\ln \, (1 \, {-}\lambda_{i} )} \,\,{\text{and}}$$

Using this test [see Eq. (7)], the null hypothesis is that there are at most *k* cointegration relationships where *λ*_*i*_ are the *m* – *k* ordered eigenvalues from the reduced rank regression.

Both the approach suggested by Toda and Yamamoto [18] and the technique developed by Johansen [17] that are used here to test for Granger causality (respectively, Granger non-causality) are able to cope with the possibility that there are dynamic interrelationships and even cointegration relationships among the time series under investigation. According to Goddard et al. [14], finding Granger causality that runs from dividends to corporate earnings would be supportive for the dividend signaling hypothesis. In contrast, the dividend smoothing hypothesis assumes that Granger causality should run from earnings to dividend payments.

## Empirical analysis

Examining the dividend time series, the non-parametric test introduced by Kruskal and Wallis [91] seems to suggest that seasonality is present. The results are reported in Table 1. This empirical finding is no major surprise, given that many European companies only pay regular dividends once a year. As a consequence, three seasonal dummy variables are added to all models estimated below.

**Table 1:** Non-parametric test for the presence of seasonality

Kruskal–Wallis		
Test stat	*df*	Prob
69.5670	3	0.000

Unit root tests indicate that both time series under investigation seem to be non-stationary variables integrated of order 1. The results of the ADF unit root tests are reported in Table 2 using the critical values that have been tabulated by Doornik [92]. Given this empirical finding, *q* = 1 using the approach suggested by Toda and Yamamoto [18]. Moreover, given this result it clearly makes sense to test for cointegration among dividends (DPS) and corporate earnings (EPS).

**Table 2:** Unit root tests

Null hypothesis: Time series have a unit root				
Exogenous: constant				
Lag length: 3 (automatic—based on SIC, maxlag = 11)				
			t-Statistic	Prob.
*ADF test*				
DPS			− 0.0418	0.9510
ΔDPS			− 139.2941	0.0001
EPS			− 2.6972	0.0792
ΔEPS			− 7.9763	0.0000

Cointegration among dividends DPS

Corporate Earnings EPS

Employing the procedure suggested by Toda and Yamamoto [18] to test for Granger causality, p has to be determined. This is done based on the Akaike information criterion. Following this approach, p is four (results are not reported to conserve space). Therefore, Eq. (3) is estimated with *p* = 4 and *q* = 1. As already noted, this approach ensures that the test statistic is asymptotically chi-square distributed. The results reported in Table 3 seem to indicate that the hypothesis of no Granger causality running from dividend payments to corporate earnings cannot be rejected. Thus, there is strong empirical evidence against dividend signaling in the European pharmaceutical industry. This interesting empirical finding fits perfectly to the idea that firms that strongly rely on internal funds to finance investment projects should consider very carefully whether the approach that is called dividend signaling in the corporate finance literature really makes sense for them because in this case, there is the danger that the increase to the amount of dividends is "financed" by lower levels of investment in the future (see, most importantly, [30]). Under these circumstances, dividends clearly are no useful leading indicator for corporate earnings. However, the empirical evidence presented in Table 3 seems to suggest that the earnings per index share time series could be helpful to forecast the dividend per index share time series. In fact, with a *p* value of 0.0735, the results reported here seem to indicate that corporate earnings Granger cause dividends (10% error level). Following Goddard et al. [14], this would imply that there is at least some empirical evidence showing that the payout policy implemented by the managers of the firms examined here seems to be guided by the strategy of dividend smoothing.

**Table 3:** Results of the Toda–Yamamoto–Granger causality tests

TY Granger causality tests			
Excluded	Chi-sq	*df*	Prob.
*Dependent variable: DPS*			
EPS	8.544898	4	0.0735
*Dependent variable: EPS*			
DPS	1.712679	4	0.7884

Given that Cheung and Lai [88] have documented that the trace test appears to be robust to an over-parametrization but can have problems with distortions estimating under-parametrized models, we again use the Akaike information criterion to determine the number of time lags to be included in the model and consider four time lags in the VECM. The results reported in Tables 4 and 5 seem to indicate that cointegration is a phenomenon of relevance examining dividend payouts and corporate earnings in the European pharmaceutical industry (1% error level). The critical values used here to test for cointegration are taken from Doornik [92]. As already noted, seasonal dummies have to be included in the models when testing for cointegration because of the strong seasonality present in the dividend time series. Interestingly, the empirical findings documented in Tables 4 and 5 imply that this empirical finding is quite robust against different deterministic trend assumptions.

**Table 4:** Johansen cointegration tests (with no deterministic trend)

Lags interval (in first differences): 1–4				
Hypothesized		Trace	5%	1%
No. of CE(s)	*p* value	Statistic	Critical Value	Critical Value
None	0.0000	47.12	20.16	24.69
At most 1	0.0670	8.50	9.14	12.53

**Table 5:** Johansen cointegration tests (with deterministic trend)

Lags interval (in first differences): 1–4				
Hypothesized		Trace	5%	1%
No. of CE(s)	*p* value	Statistic	Critical Value	Critical Value
None	0.0033	33.74	25.73	30.67
At most 1	0.2913	7.64	12.45	16.22

**Fig. 3:**
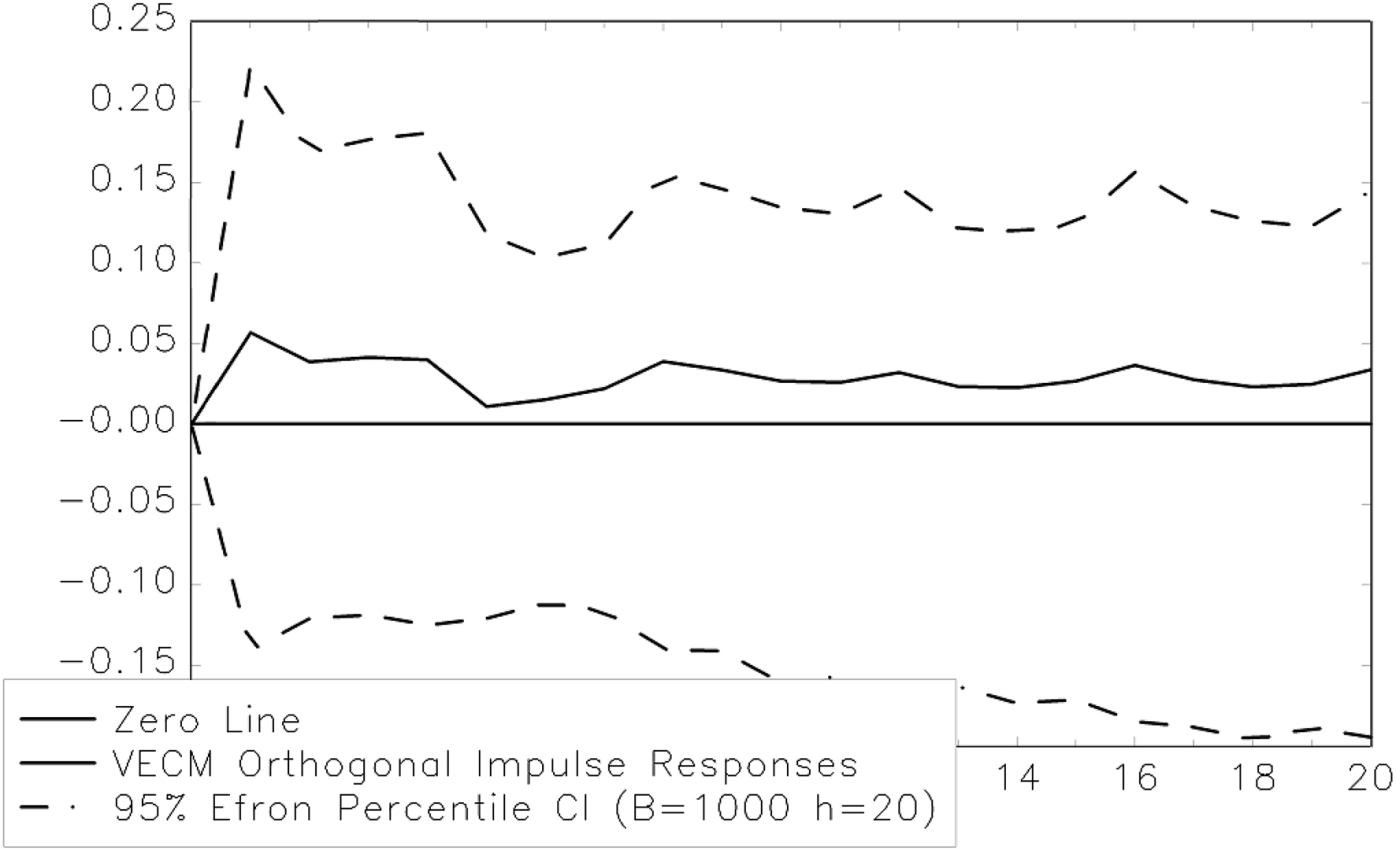
Impulse response function (exogenous shock DPS)

**Fig. 4:**
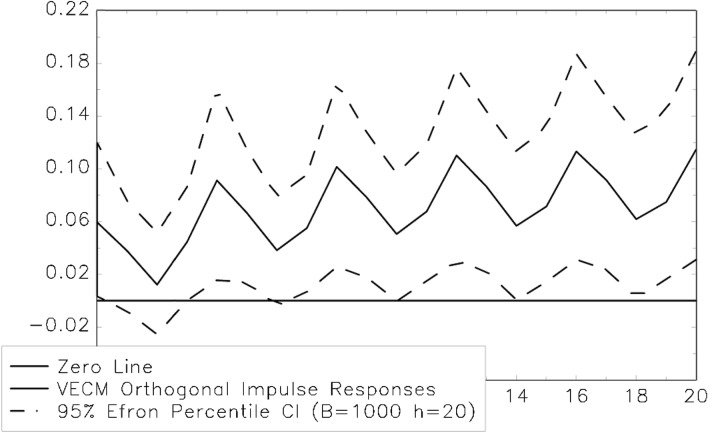
Impulse response function (exogenous shock EPS)

Thus, there is clear empirical evidence for the existence of one cointegration relationship between dividend payouts and corporate earnings in the European pharmaceutical industry. This finding also has implications searching for Granger causality (see, for example, [16, 93]). In fact, the existence of a cointegration relationship among two time series implies that there has to be either (I) unidirectional Granger causality running from the first to the second variable, (II) unidirectional Granger causality running from the second to the first variable or (III) bidirectional Granger causality among the two variables under investigation. This fact clearly could strengthen the relatively weak empirical evidence for dividend smoothing reported in Table 3. Therefore, the VECM should also be used to test for Granger causality. The technique of impulse response analysis is employed to do so. This is a very popular approach in the field of applied econometrics (see, for example, [13, 94]). The confidence intervals (5% error level) are bootstrapped using the Efron approach with 1000 replications [95]. The results are reported in Figs. 3 and 4.

Again, there is no statistically significant lagged reaction of corporate earnings to an exogenous shock to dividend payouts—and therefore, no empirical evidence for Granger causality running from dividends to earnings (see Fig. 3). Thus, using this approach also produces results that are not compatible with the predictions of the dividend signaling hypothesis. However, there is additional empirical evidence for dividend smoothing in the European pharmaceutical industry. In fact, there is a statistically significant (5% error level) lagged reaction of dividend payments to an exogenous shock to corporate earnings. This interesting result is indeed a clear indication of the existence of Granger causality running from corporate earnings to dividend payouts. Consequently, using a different empirical research strategy also produces unmistakable evidence against dividend signaling in the European pharmaceutical industry and shows quite clearly (this time on the 5% error level) that the firms smooth their dividend payments. Given the special situation of business enterprises that belong to this economic sector (namely high R&D expenditures with the corresponding risks and a considerable possibility of being involved in litigation), these empirical results probably are no major surprise. Nevertheless, these findings are very interesting, as there has been little empirical research on this question so far. Moreover, the empirical evidence reported here also seems to be supportive of the model explaining dividend smoothing that has been proposed by Karpavičius [96], who has argued that it is necessary to simultaneously consider the operating, financial and investment decisions of managers to illustrate the behavior of “real” firms in a more appropriate way. This model predicts that firms should try to keep their dividends stable to maximize their shareholders' wealth. It does not try to explain the existence of the practice of dividend smoothing with information asymmetries or agency issues but with the intention of managers to reduce the likelihood of dividend omissions or cuts that arise from their commitment to ensure the availability of certain amounts of cash each period. This idea clearly helps to explain the empirical finding (see, most importantly, [97]) that many firms decide to smooth their dividend payments. We add to this literature by examining data from an industry where internal financing is of central importance. Therefore, not being able to find indications for dividend smoothing in the pharmaceutical industry clearly would, for example, be quite problematic for the theoretical model of dividend smoothing suggested by Karpavičius [96].

## Conclusion

From the perspective of corporate finance theory, the high R&D expenditures of the pharmaceutical industry—which for example, may affect the payout policy of firms because of the higher likelihood of being sued in costly cases of patent litigation—make an empirical study of dividend payments in this sector of the economy particularly interesting. Generally speaking, Malm and Kanuri [12] have argued that pending lawsuits could reduce the willingness of firms to pay dividends. Therefore, they have concluded that an increased litigation risk should result in a more conservative dividend policy of firms. Phrased somewhat differently, managers that fear the possible burden of high legal costs in the future might show less propensity to pay out funds as dividends today that could be quite helpful tomorrow. With regard to two important theories of dividend determination that are very popular in the field of corporate finance—namely dividend signaling and dividend smoothing—such considerations could imply that firms belonging to the pharmaceutical industry might prefer dividend smoothing to dividend signaling. Dividend smoothing can be interpreted as dividend signaling with precaution because firms that follow this strategy when implementing their payout policy want to reduce the need to lower the future volume of dividend payments fearing that financial markets could overreact to the news that a firm has been forced to reduce or even omit its payout of regular dividends.

The empirical evidence presented above indicates that the European pharmaceutical industry has smoothed its dividends in the period 2002–2020. In fact, there is clear empirical evidence for Granger causality running from corporate earnings to dividend payments.These findings add to a number of recent empirical studies of dividend policy issues (see, for example, [74, 98]) that examine data from specific economic sectors. Given the high R&D expenditures common among firms in the pharmaceutical industry, the results reported here should be of particular interest. As discussed above, they might, for example, have implications for researchers that are interested in the relationship between litigation risk and the payout policy of potentially or actually sued firms. For the pharmaceutical industry, Lakdawalla [9] observes shifts in the R&D from small molecule drugs toward biologicals and also an increased concentration of R&D in smaller companies. The possible effects of these trends on the dividend policy of pharmaceutical companies might provide interesting results in future studies. Moreover, it might be a good idea to also examine data from non-European countries.
